# Risk Assessment of Arsenic in Rice Cereal and Other Dietary Sources for Infants and Toddlers in the U.S.

**DOI:** 10.3390/ijerph13040361

**Published:** 2016-03-25

**Authors:** Tomoyuki Shibata, Can Meng, Josephine Umoren, Heidi West

**Affiliations:** 1 Public Health Program, Northern Illinois University, DeKalb, IL 60115, USA; z1653061@students.niu.edu; 2 Global Environmental Health LAB, Not-for-Profit Organization, Brooklyn, NY 11206, USA; hwest@gehlab.org; 3 Division of Statistics, Northern Illinois University, DeKalb, IL 60115, USA; 4 Nutrition & Dietetics Program, Northern Illinois University, DeKalb, IL 60115, USA; jxu1@niu.edu

**Keywords:** risk assessment, infant and toddler health, arsenic, rice cereal, food standard

## Abstract

Currently, there are no set standards or quantitative guidelines available in the U.S. for arsenic levels in rice cereal, one of the most common first solid foods for infants. The objective of this study was to evaluate whether the detected levels of inorganic arsenic (As_i_) in rice cereal in the U.S. market are safe for consumption by infants and toddlers. A risk assessment was conducted based on literature reviews of the reported As_i_ in rice cereal from the U.S. Food and Drug Administration’s (FDA) survey and the recommended daily intake of rice cereal by body weight, for infants and toddlers between four and 24 months old. As a part of risk management, a maximum contaminant level (MCL) for As_i_ in rice cereal was computed considering overall exposure sources including drinking water, infant formula, and other infant solid foods. Hazard quotients (HQs) for acute and chronic exposures were calculated based on the U.S. Agency for Toxic Substances and Disease Registry’s (ATSDR) Minimal Risk Level (MRL)_acute_ (5.0 × 10^−3^ mg/kg/day) and MRL_chronic_ (3.0 × 10^−4^ mg/kg/day). A cancer slope or potency factor of 1.5 mg/kg/day was used to predict an incremental lifetime cancer risk (ILCR). Exposure assessment showed that the largest source of As_i_ for infants and toddlers between four and 24 months old was rice cereal (55%), followed by other infant solid food (19%), and drinking water (18%). Infant formula was the smallest source of As_i_ for babies (9%) at the 50th percentile based on Monte Carlo simulations. While HQ_acute_ were consistently below 1.0, HQ_chronic_ at the 50 and 75th percentiles exceeded 1.0 for both rice cereal and total sources. ILCR ranged from 10^−6^ (50th) to 10^−5^ (75th percentile). MCLs for As_i_ in rice cereal ranged from 0.0 (chronic) to 0.4 mg/kg (acute exposures).

## 1. Introduction

There is increasing concern regarding arsenic contaminants in rice cereal, which is one of the most common first solid infant foods in the U.S. [1,2]. Arsenic is commonly recognized as a toxic metalloid that naturally occurs in soil and groundwater, and is known to accumulate in rice at higher levels than in other crops [3,4,5,6,7,8,9,10,11,12,13,14]. A recent survey conducted by the U.S. Food and Drug Administration (FDA) showed that rice cereal products sold in the U.S. market contained arsenic ranging from 0.050 to 0.723 mg/kg [15,16]. The guidelines for arsenic in food in the U.S. apply only to byproducts of animals treated with veterinary drugs (e.g., permissible levels of arsenic from 0.5 ppm in eggs and uncooked edible tissues of chickens and turkeys to 2 ppm in certain uncooked edible byproducts of swine) [17]. Currently, there is no direct standard in the U.S. to evaluate whether the amounts of arsenic in rice cereal are within safe levels for consumption by infants and toddlers between the ages of four and 24 months [1,2,13,16,18].

Arsenic in rice cereal sold in the U.S. was reported to consist of 37% organic form (As_o_) including monomethylarsonic acid (MMA) and dimethylarsinic acid (DMA), and 63% inorganic form (As_i_) that ranged from 0.023 to 0.283 mg/kg [15,16]. The toxicity of arsenic varies depending on its forms and As_o_ is known to be less harmful to human health compared to As_i_ [13]. MMA and DMA have been widely used as pesticides and herbicides in the U.S. [13]. As_i_ exposure has comparatively harmful effects on human health including carcinogenic and/or non-carcinogenic effects on many different organs such as the skin, gastrointestinal tract, bladder, liver, and lungs [13]. Accordingly, this public health study focuses on As_i_ and its toxic contents.

Recently, the Joint Food and Agriculture Organization and the World Health Organization (FAO/WHO) Expert Committee on Food Additives proposed a maximum level (ML) of As_i_ of 0.2 mg/kg for polished rice [16,19]. While this ML of As_i_ 0.2 mg/kg has not yet been adopted by U.S. regulatory agencies, Signes-Pastor *et al.* [16] raised concern that some rice cereals could exceed this proposed reference level.

Despite the fact that the potential health risks from rice cereal consumption are not well understood, caregivers have been told to reduce the amount of rice cereal consumed by infants and toddlers [1]. Babies are presumably exposed to As_i_ not only from rice cereal, but also from water and other dietary sources including infant formula, purees, and 2nd/3rd stage foods [20,21]. How much rice cereal contributes to the overall As_i_ exposure has not been evaluated quantitatively to date. It is important to improve the scientific basis to assist decision makers in determining necessary actions to immediately protect babies from potential As_i_ exposure [12,16]. The objective of this study was to assess risk of As_i_ exposure from rice cereal as well as from the overall exposure sources in the U.S. This study also attempted to compute a reference level or Maximum Contaminant Level (MCL) for rice cereal considering overall sources and human factors (e.g., diet and body size) in the U.S. in order to compare this MCL to the proposed international guideline of 0.2 mg/L.

## 2. Methods

This study followed a risk assessment framework consisting of hazard identification, dose-response, exposure assessment, and risk characterization followed by risk management. This is a practical approach designed to provide decision-makers with valuable information based on the limited data currently available [22]. Hazard identification was described in the introduction section. For dose-response, this study referred to the U.S. Agency for Toxic Substances and Disease Registry’s (ATSDR) Minimal Risk Levels (MRLs) for acute oral consumption at 5.0 × 10^−3^ mg/kg/day and chronic oral consumption at 3.0 × 10^−4^ mg/kg/day as safe doses [23]. The U.S. ATSDR’s MRL for chronic exposure is the same as the U.S. Environmental Protection Agency’s (EPA) Reference Dose (RfD) for inorganic arsenic, which was used to establish the National Primal Drinking Water Regulations (NPDWRs) [13,24,25]. A slope factor of 1.5 (mg/kg/day)^−1^ was used to compute incremental lifetime cancer risk of arsenic in baby’s diets [24]. Quantitative procedures for exposure assessment, risk characterization, and risk management are described in the following sections.

### 2.1. Exposure Assessment

A range of As_i_ doses for infants and toddlers between four and 24 months who consume water, infant formula, rice cereal, and other infant solid foods such as puree and 2nd/3rd stage foods was calculated considering changes in diet and body weight by age (Equation (1)):

(1) $$ADD_{x,t}=C_{x}\cdot V_{x,t}\cdot BW_{t}^{-1}$$

where *ADD_x,t_* is an average daily dose of As_i_ from a specific dietary source *x*, at specific ages *t*, (from four to 24 months); *C_x_* is a concentration of As_i_ in a specific source *x*: water (*C_w_*), infant formula (*C_if_*), rice cereal (*C_rc_*), and other infant solid foods (*C_o_*) containing fruits, vegetables, and meats; *V_x_* is an amount of specific food *x*: water (*V_w,t_*), infant formula (*V_if,t_*), rice cereal (*V_rc,t_*), and other infant solid food including fruits (*V_f,t_*), vegetables (*V_v,t_*), and meats (*V_m,t_*) consumed by babies at the specific age *t*; *BW_t_* is specific body weight at age *t*. Chronic daily intake (CDI) was calculated as a mean of *ADD_t_* between four and 24 months (Equation (2)). Lifetime average daily dose (LADD) was estimated assuming that As_i_ exposure from rice cereal occurred only between four and 24 months over the lifetime (Equation (3)):

(2) $$CDI=\left(\frac{1}{21}\sum_{4}^{24}ADD_{x,t}\right)$$

(3) $$LADD=CDI\cdot \frac{ED}{AT}$$

where *21* is a total number of data sets (from 4 to 24 months); *ADD_x_,_t_* was calculated based on Equation (1); *ED* is an exposure duration (*ED* = 20 months = 620 days); *AT* is the average time (A*T* = 70 years is typical used for risk assessment = 25,550 days). While a LADD based on such a short-term exposure might be unrealistic, this study referred to an earlier study that estimated the LADD of arsenic and cancer risk through childhood exposure (1–6 years old) [26].

Table 1 summarized values and assumptions for As_i_ levels in multiple exposure sources. Human factors (e.g., age specific body weight and intake volumes) were summarized in Table 2. The types and amounts of liquid and solid foods consumed by infants vary depending on age [27,28]. The amounts of direct and indirect water intake (*V_w_*) and milk consumption (*V_if_*) by different age groups were obtained from the *Child-Specific Exposure Factors Handbook* [28]. The amounts of infant formula, rice cereal, fruits, vegetables, and meats consumed per feeding were obtained from Fox *et al.* [29] and U.S. EPA [28]. The volume of infant formula consumed was calculated based on the dry weight (e.g., 1 fluid ounce (oz.) of formula was converted to 4.3 g of dry formula). Infants were assumed to start rice cereal consumption at four months of age and then hot rice cereal from 12 to 24 months. The amount of rice cereal consumed was calculated based on the dry weight. For dry cereal, one tablespoon (tbsp.) and one cup of dry cereal were assumed to be 4.6 g and 73.6 g, respectively, based on weighing a popular product sold in the U.S. Infants were assumed to start eating fruits and vegetables at four months old and meats at six months old [30]. For fruits and vegetable, 1 tbsp. and 1 cup were converted to 16.3 g and 26.08 g, respectively. For meats, 1 tbsp. and 1 oz. were converted to 16.3 g and 28.35 g, respectively. The average body weights were calculated according to sex and age from four to 24 months old [28].

In order to provide a range of possible As_i_ exposures and associated risk, Monte Carlo simulations were performed one million times for each computation using Crystal Ball software (Oracle, Redwood Shores, CA, USA) based on limited available data.

### 2.2. Risk Characterization

After integrating information from the proceeding steps of the exposure assessment, health risks as a result of acute and chronic exposure were characterized based on Equations (4)–(6):

(4) $$HQ_{acute}=ADD_{max}\cdot MRL_{acute}^{-1}$$

(5) $$HQ_{chronic}=CDI\cdot MRL_{chronic}^{-1}$$

where *HQ_acute_* and *HQ_chronic_* are hazard quotients for acute and chronic exposures, respectively; *ADD_max_* is the largest *ADD_t_* observed between four and 24 months in the exposure assessment; CDI was chronic daily intake calculated in the exposure assessment; and *MRL_acute_* and *MRL_chronic_* are the U.S. ATSDR’s MRLs of 5.0 × 10^−3^ mg/kg/day for acute and 3.0 × 10^−4^ mg/kg/day for chronic consumptions, respectively [23].

If the HQ is calculated to be equal to or less than 1, then no adverse health effects are expected as a result of exposure. Incremental lifetime cancer risk (ILCR) was estimated based on Equation (6).

(6) ILCR=CSF·LADD

where *ILCR* is an incremental lifetime cancer risk as a result of exposures occurring between four and 24 months of age; *CSF* is a cancer slope or potency factor (*CSF* = 1.5 (mg/kg/day)^−1^ [24]; and *LADD* is a lifetime average daily dose calculated in the exposure assessment.

### 2.3. Risk Management

Currently, there is no guideline for As_i_ in rice cereal in the U.S. Thus, maximum contaminant levels (MCL) for As_i_ in rice cereal, which would result in ATSDR’s MRL_acute_ and MRL_chronic_, were computed based on the Equations (7) and (8), respectively with consideration of overall As_i_ exposure. The MCLs, which are used for the NPDWRs, are the maximum allowable levels of chemicals considering public health and acceptable risk:

(7) $$MCL_{rc,acute}=\left(\sum_{4}^{24}\left((MRL_{acute}\cdot BW_{t}-(MCL_{w}\cdot V_{w,t}+C_{if}\cdot V_{if,t}+C_{o}\cdot (V_{fr,t}+V_{v,t}+V_{m,t})))\cdot V_{c,t}^{-1}\right)\right)\cdot 21^{-1}$$

(8) $$MCL_{rc,chronic}=\left(\sum_{4}^{24}\left((MRL_{chronic}\cdot BW_{t}-(MCL_{w}\cdot V_{w,t}+C_{if}\cdot V_{if,t}+C_{o}\cdot (V_{fr,t}+V_{v,t}+V_{m,t})))\cdot V_{c,t}^{-1}\right)\right)\cdot 21^{-1}$$

where *MCL_rc, acute_* and *MCL_rc, chronic_* are reference levels for As_i_ in rice cereal for acute and chronic exposures, respectively. *MCL_w_* is the maximum contaminant level for arsenic (0.010 mg/L) in drinking water based on the NPDWR. For the exposure assessment, the range used was 0 to 0.010 mg/L. However, the more conservative level of *MCL_w_* 0.01 mg/L was used for computation of the *MCR*_rc_. The total number of data sets for infants and toddlers from 4 to 24 months old is 21.

## 3. Results

**Figure 1:**
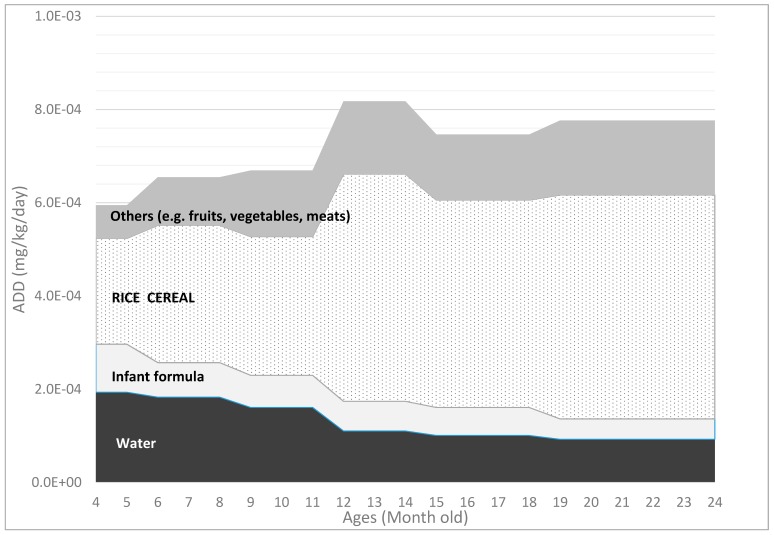
Average daily doses (ADDs) of As_i_ (mg/kg/day) from water and dietary sources for infants and toddlers between four and 24 months old.

Table 3 summarizes the *ADD_x,t_* of As_i_ from water and dietary sources for babies at the 25th (low) 50th (median) and 75th (upper) percentiles based on the Monte Carlo simulations. As_i_ doses from rice cereal and other solid food increased by age, while the doses from drinking water and infant formula decreased comparatively (Figure 1). The largest source of As_i_ for infants and toddlers was rice cereal (55% based on CDI or the mean of ADD between four and 24 months at the 50th percentile), followed by other infant solid (19%), and drinking water on average (18%). Infant formula was the smallest source of As_i_ for babies (9%).

*HQ_acute_* from rice cereal as well as from total sources were below 1.0 at the lower and upper percentiles (Table 4). HQ_chronic_ at the 50 and 75th percentiles exceeded 1.0 for both rice cereal and total sources while the values at the 25th percentile was below 1.0. ILCR ranged from 10^−6^ at the 25th percenitle to 10^−5^ at the 75th percentile for both rice cereal and total sources.

*MCL_rc,__acute_* was calucated to be As_i_ 4.1 × 10^−4^ mg/g (or 0.4 mg/kg) based on the 25th percentile, which is more protective than the refence base on the 50th percentile. *MCL_rc,__chronic_* showed negative values since *HQ_chronic_* exceeded 1.0. Alternatively, *MCL_rc,__chronic_* was set as 0.0 mg/kg.

## 4. Discussion

As_i_ contents in rice cereals have been well documented. This study contributed to scientific evidence on As_i_ doses for infants and toddlers from four to 24 months old through rice cereal, drinking water, and other solid foods for infants in the U.S. The simulations in this study suggest that some babies could have been exposed to larger doses while the computed As_i_ doses at the 50th percentile were within the same range as an earlier study that showed a baby’s As_i_ to be 1.4 × 10^−4^ mg/kg/day in the U.S. [31].

Solid food, including rice cereal and others (e.g., purees and 2nd/3rd stage foods) were estimated to account for 75% of overall As_i_ doses, which was consistent with Xue *et al.* [31], suggesting that the U.S. population is exposed to more As_i_ through solid foods than from drinking water, which has been regulated for arsenic. Infant formula contributes the least to the overall inorganic arsenic exposure. This finding was consistent with earlier studies indicating that arsenic exposure from milk was less of a concern than exposure from other sources [32,33]. It must be noted that infants’ and toddlers’ inorganic arsenic exposures were based on an assumption that they eat rice cereal on a daily basis. Actual doses for individuals vary if they do not eat rice cereal regularly or eat additional rice products (e.g., baby rice or rice cracker).

One of the significant findings of this study was that rice cereal could account for over a half of As_i_ exposure for babies in the U.S if they consumed it on daily basis. This assessment is based on the ATSDR’s MRLs safe doses for acute and chronic exposures which are derived from the non-observed adverse health levels divided by uncertainty factors considering the effects of toxic chemicals in the most risky populations including infants, elderly, and nutritionally or immunologically compromised people [13,24]. This study shows that the risk associated with rice cereal is below minimal or acceptable level in terms of acute effects. On the other hand, the simulations suggest that those infants and toddlers whose As_i_ dose levels were at the upper percentiles could have more than a minimal risk of non-carcinogenic chronic effects.

The computed incremental lifetime cancer risk from rice cereal as well as the overall exposure during infancy in this study was 10^−5^, which was compatible with acceptable risk suggested by the World Health Organization (10^−5^) and the U.S. EPA (10^−4^ to 10^−6^) for carcinogens in drinking water [34]. This study characterized risk based on arsenic exposure models for the relatively short exposure of infancy over average time. Possible effects were less likely homogeneously distributed over a long lifetime, which is one of limitations of the current risk assessment. There has been discussion on an alternative cancer slope of 3.6 (mg/kg/day)^−1^ [35]. An incremental lifetime cancer risk based on this alternative value will be 2.4 times higher than the risk based on the current EPA’s suggested slope of 1.5 (mg/kg/day)^−1^, which was used in this study. Although such a difference was within our predicted values based on Monte Carlo simulations, a future study may need to use the alternative slope once it is approved by the U.S. EPA.

Currently, there is no guideline for arsenic content in baby food including infant formula, rice cereal, and other infant solid food in the U.S. Although the study concurs that caregivers do not need to remove rice cereal from a baby’s diet immediately, considering its nutrition benefits [18], it will be important to establish a guideline for rice cereal in order to reduce potential risk of chronic effects of As_i_ exposure. The JECFA suggests the maximum level of As_i_ in polished rice to be 0.2 mg/kg [19], which is exactly between 0.0 mg/kg for chronic and 0.4 mg/kg for acute effects computed in this study. The maximum contaminated level (MCL) of 0.0 mg/kg is not realistic, but can be set as a maximum contaminant level goal (MCLG). As_i_ contents in rice cereal from the U.S. FDA survey and others reports [1,15,16,18] were all below 0.4 mg/kg while some exceeded 0.2 mg/kg. Risk management in this study supports the U.S. regulatory agencies consideration for adopting the JECFA’s 0.2 mg/kg [19] maximum level. We recommend that the food industry voluntarily monitor levels of ASI based on the suggested value of 0.2 mg/kg in order to ensure that their projects are safe for infants and toddlers.

## 5. Conclusions

Assessing the safety of baby food is challenging. While it has limitations, risk assessment can provide decision-makers with useful quantitative information on difficult subjects. The current risk assessment was unique, but it will be necessary to gather more data on arsenic in foods and conduct an epidemiologic study in order to have a more accurate risk assessment validate the findings from this study. Further improvement of the scientific basis is important to ensure that public health will be protected, especially for vulnerable populations such as infants and toddlers.

## Figures and Tables

**Table 1:** Values and assumptions of As_i_ in water (*_w_*), infant formula (_if_), rice cereal (*_rc_*), and other infant solid food (_o_) in the Monte Carlo simulations.

Parameter	Distribution, Values, References
*C_w_* (mg/L)	Uniform: from the maximum contaminant level goal (0 mg/L) to the MCL (0.010 mg/L) for arsenic in the NPDWRs [25]
*C_if_* (mg/L)	Normal: mean (7.48 × 10^−6^) with a standard deviation (6.12 × 10^−7^). with an assumption that detected arsenic was exclusively As_i_ [20]
*C_rc_* (mg/g)	Triangular: minimum (2.30 × 10^−5^), likeliest (9.10 × 10^−5^), and maximum (2.83 × 10^−4^) [15,16]
*C_o_* (mg/g)	Normal: mean (4.6 × 10^−6^) with a standard deviation (5.6 × 10^−6^). C_o_ was combined from purees and 2nd/3rd stage foods including fruits, vegetables, and meats with an assumption of that 81.5% of total arsenic was As_i_ [20]

**Table 2:** Values and assumptions for age specific body weights (*BW*) and ingestion volumes (*V*) of water (*_w_*), infant formula (*_if_*), rice cereal (_rc_), fruits (*_fr_*), vegetables (*_v_*), and meats (*_m_*) in the Monte Carlo simulations.

Infant’s Age	Body Weight					Intake Voumes per Day	
(Month)	*BW* (kg)	*V_w_* (L/Day)	*V_if_* (g/Serving) × (Servings/Day)	*V_rc_* (g/Day)	*V_fr_* (g/Serving) × (Serving/Day)	*V_v_* (g/Serving) × (Serving/Day)	*V_m_* (g/Serving) × (Serving/Day)
	Normal *	Triangular ^Ɨ^	Normal	Normal	Normal	Normal	Normal
4–5	6.95 ± 0.85	0, 0.148, 0.924	21.5 ± 9.7 × 4–5 ^ǂ^	14.3 ± 16.1	58.7 ± 77.4 × 1–2	61.9 ± 81.4 × 1–2	
6–8	7.95 ± 1.08	0, 0.218, 0.885	22.8 ± 11.4 × 3–4	20.7 ± 17.1	76.6 ± 47.7 × 1–2	94.5 ± 69.4 × 1–2	25.5 ± 120.7 × 1–2
9–11	9.03 ± 1.05	0, 0.218, 0.885	24.1 ± 10.1 × 3–4	23.9 ± 21.7	94.5 ± 72.6 × 1–2	91.3 ± 85.5 × 2–3	22.7 ± 37.2 × 1–2
12–14	9.93 ± 1.08	0, 0.188, 0.624	24.5 ± 22.4 × 3–4	44.2 ± 70.9	104.3 ± 100.5 × 1–2	104.3 ± 100.5 × 2–3	34.0 ± 32.8 × 1–2
15–18	10.90 ± 1.50	0, 0.188, 0.624	25.4 ± 52.4 × 3–4	44.2 ± 70.9	130.4 ± 138.2 × 1–2	104.3 ± 138.2 × 2–3	36.9 ± 40.1 × 1–2
19–24	11.85 ± 1.38	0, 0.188, 0.624	20.2 ± 75.4 × 3–4	51.5 ± 65.8	156.5 ± 134.0 × 1–2	104.3 ± 93.3 × 2–3	36.9 ± 35.5 × 1–2

* indicates mean ± standard deviation (normal distribution). **^Ɨ^** 10th, 50th, 90th percentiles (triangular distribution). ^ǂ^ × indicates multiplication with range of frequencies. Body weight and water consumption (V_w_) were based on U.S. EPA [28] and other ingestion volumes were based on Fox *et al.* [29].

**Table 3:** Average daily doses (*ADD_x,t_*) of As_i_ (mg/kg/day) from drinking water and dietary sources.

Age (Month)	Drinking Water			Infant Formula			Rice Cereal			Other Solid Food			Total Sources		
	25th	50th	75th	25th	50th	75th	25th	50th	75th	25th	50th	75th	25th	50th	75th
4–5	3.2 × 10^−5^	1.9 × 10^−4^	3.8 × 10^−4^	7.1 × 10^−5^	1.0 × 10^−4^	1.4 × 10^−4^	5.1 × 10^−5^	2.3 × 10^−4^	4.6 × 10^−4^	0.0	7.1 × 10^−5^	2.2 × 10^−4^	2.1 × 10^−4^	5.9 × 10^−4^	1.2 × 10^−3^
6–8	3.1 × 10^−5^	1.8 × 10^−4^	3.4 × 10^−4^	4.8 × 10^−5^	7.4 × 10^−5^	1.0 × 10^−4^	1.2 × 10^−4^	2.9 × 10^−4^	5.3 × 10^−4^	0.0	1.0 × 10^−4^	2.9 × 10^−4^	2.5 × 10^−4^	6.5 × 10^−4^	1.3 × 10^−3^
9–11	2.7 × 10^−5^	1.6 × 10^−4^	3.0 × 10^−4^	2.4 × 10^−5^	6.9 × 10^−5^	9.1 × 10^−5^	1.1 × 10^−4^	3.0 × 10^−4^	5.5 × 10^−4^	1.0 × 10^−5^	1.4 × 10^−4^	3.4 × 10^−4^	2.4 × 10^−4^	6.7 × 10^−4^	1.3 × 10^−3^
12–14	1.9 × 10^−5^	1.1 × 10^−4^	2.0 × 10^−4^	0.0	6.4 × 10^−5^	1.0 × 10^−4^	0.0	4.9 × 10^−4^	1.1 × 10^−3^	1.3 × 10^−5^	1.6 × 10^−4^	3.7 × 10^−4^	8.7 × 10^−5^	8.2 × 10^−4^	1.8 × 10^−3^
15–18	1.7 × 10^−5^	1.0 × 10^−4^	1.8 × 10^−4^	0.0	6.0 × 10^−5^	1.5 × 10^−4^	2.0 × 10^−6^	4.4 × 10^−4^	1.0 × 10^−3^	8.7 × 10^−7^	1.4 × 10^−4^	3.8 × 10^−4^	4.8 × 10^−5^	7.5 × 10^−4^	1.7 × 10^−3^
19–24	1.6 × 10^−5^	9.3 × 10^−5^	1.7 × 10^−4^	0.0	4.4 × 10^−5^	1.6 × 10^−4^	6.3 × 10^−5^	4.8 × 10^−4^	1.0 × 10^−3^	1.9 × 10^−5^	1.6 × 10^−4^	3.7 × 10^−4^	1.2 × 10^−4^	7.8 × 10^−4^	1.7 × 10^−3^

Note: Negative values from Monte Carlo simulations with large standard distributions were converted to 0.0.

**Table 4:** Summary of mean As_i_ doses and associated risk characteristics.

As_i_ Dose and Risk	Rice Cereal			Total Sources		
	25th	50th	75th	25th	50th	75th
ADD_max_ (mg/kg/day)	1.2 × 10^−4^	4.9 × 10^−4^	1.1 × 10^−3^	2.5 × 10^−4^	8.2 × 10^−4^	1.8 × 10^−3^
CDI (mg/kg/day)	5.6 × 10^−5^	4.0 × 10^−4^	8.5 × 10^−4^	1.5 × 10^−4^	7.3 × 10^−4^	1.6 × 10^−3^
LADD (mg/kg/day)	1.3 × 10^−3^	9.5 × 10^−6^	2.0 × 10^−5^	3.5 × 10^−6^	1.7 × 10^−5^	3.7 × 10^−5^
HQ_acute_	0.02	0.10	0.23	0.05	0.16	0.37
HQ_chronic_	0.19	1.33	2.83	0.49	2.42	5.17
ILCR	2.0 × 10^−6^	1.4 × 10^−5^	3.0 × 10^−5^	5.2 × 10^−6^	2.6 × 10^−5^	5.5 × 10^−5^
